# Alterations of the Biochemical Pathways of Plants by the Air Pollutant Ozone: Which are the True Gauges of Injury?

**DOI:** 10.1100/tsw.2007.19

**Published:** 2007-03-21

**Authors:** Robert L. Heath

**Affiliations:** Department of Botany and Plant Sciences, University of California, Riverside, CA, USA

**Keywords:** ozone injury, wounding response, anitoxidants, hydrogen peroxide, Rubisco gene regulation, plants

## Abstract

Plant strategies to survive ozone stress include exclusion or tolerance of ozone. If these processes fail, past observations of ozone injury have indicated many physiological and metabolic changes then occur; most of these changes are likely to have been initiated at the level of gene expression, suggesting signal transduction. In the last decade considerable understanding of the biochemical process within plants has been developed. Currently there are several hypotheses regarding a response of plants to ozone fumigation: [1] membrane dysfunction and alteration of purpose; [2] stress ethylene interactions; [3] impairment of photosynthesis via changes in Rubisco levels and the guard cells so that the stomata do not track correctly the environment; [4] antioxidant protection through metabolites and enzyme systems to reduce the oxidant load; and [5] general impairment or disruption of metabolic pathways.

Many believe that free radicals and other oxidative products, formed in plant leaves under ozone exposure, are responsible for much of the spread of the biochemical alterations. There are obvious chemicals that may account for the changes that are observed, such as hydrogen peroxide. Once the ozone enters the tissue, evidence suggests the first line of defense is a range of antioxidants, such as ascorbate, glutathione peroxidase, superoxide dismutase, and catalase. If overwhelmed, subsequent events occur which are highly suggestive of systemic acquired resistance. Furthermore, other defensive indicators, such as salicylic acid and jasmonic acid, tend to increase, but more slowly than ethylene, and spread their signaling effects more widely in the plant.

The primary set of metabolic reactions that ozone triggers is thought to be “wounding“ responses with a secondary response of senescence. The dramatic strides in understanding the genetic make-up of plants, gene control, and signal transduction/control over the last few years will only accelerate in the future. We need now to have an understanding of those events that can be translated into more detailed schemes of how ozone alters much of the basic metabolism of plants and how plants counteract or cope with ozone. What is now known about how varied biochemicals and their pathways are changed upon ozone exposure will be discussed.

In simple terms, we can describe how ozone changes the life of the plant by splitting the sequential influence into four areas (see Fig. 1): (1) ozone exposure, which includes the amount of $O_3$ in the atmosphere, the duration and shape of exposure, and the exposure frequency; (2) the entrance of the ozone into the tissues and cells of the plant, which includes stomatal and cuticular behavior, water potential of leaf, and apoplastic antioxidants; (3) the cellular events and the changes to metabolism; and (4) the actual observable responses of the plant, such as the loss of productivity, generation of chlorosis or necrosis, temporal shifts in development, and its ability to tolerate other stresses. The actual connection between area 2 and area 4, the cellular and biochemical responses to either ozone or its products, are the subject of this paper (see Table 1).

First, the movement of ozone into the apoplastic region will be similar to the movement of $CO_2$ into the cell (see Fig. 2). There is a diffusive or boundary layer next to the cuticle, followed by the stomatal pathway into the intracellular space of the leaf up to and through the apoplastic or wall region. With $CO_2$, we can calculate that movement and concentration of $CO_2$ well, with defined concentrations next to the plasma membrane. $CO_2$ does not react with much until it enters the cell and integrates into normal metabolism. This is not the case with ozone. It is highly reactive and can interact with components of the cuticle (although not vigorously) and with cell wall material from the stomatal and the apoplastic region. Thus, we really do not know the concentration of ozone that could be present at the cellular membrane (shown as the dark region of uncertainty in Fig. 2). Since the reactions of ozone with water depend on its concentration as well as pH and the present of other compounds, such as reducing biochemical (i.e., ascorbate) and metals[1], we cannot currently speak with any confidence to the types of reactions that may be occurring.

There are two points to be made for ozone movement into the tissue. The first is that ozone clearly alters the stomatal responses; in general, ozone exposure causes a partial closure of the stomata[2] and this will limit the amount of assimilation of $CO_2$ into carbohydrates[3], although there is evidence accumulating that stomata may become sluggish in response or even slightly open in the presence of low concentrations of ozone[4]. We still are not clear on how ozone reacts with the guard cells. However, less assimilation means less carbohydrate present for use, storage, and transport. Unfortunately, sucrose acts as a powerful signal[5,6] to many of the metabolic pathways that will change their pool sizes and alter the speed of carbon flow through the pools. When exposure causes a metabolic event, what it is due to — actual direct interference or a lessen sugar supply — is hard to understand. This problem has continued to plague research efforts.

Second, while we do not know exactly what compounds are formed, we have a strong notion that hydrogen peroxide is one of them. There have been several reports of the production of $H_2O_2$ in the cell structure of plants; see Pellinen et al.[9] and Fig. 3. There are other possible reactive species[1], such as superoxide, hydroxyl radical, and peroxyl radical. Furthermore, ERP signals due to free radicals[10] have been discovered under $O_3$ exposure, but the chemical species causing those signals are not easily identified.

Most of the evidence suggests that $H_2O_2$ builds up within the apoplastic space, where extracellular ascorbate exists[9,11]. It is believed that this specific pool of ascorbate reacts with ozone or its products. That reaction reduces the amount of $O_3$ present at the membrane, but it has a cost of ascorbate loss. Mudd[12] argued that ozone can easily break the double bond within ascorbate, effectively destroying that particular molecule. To replenish that particular pool of ascorbate under those conditions would take a great amount of energy (even starting from glucose). If $H_2O_2$ is the reactive species, then ascorbate would be oxidized to dehydroascorbate and only one NADPH would be required to reduce that molecule back to ascorbate. The energy cost would be relative small, but not zero, under those conditions. So the rate of $O_3$ entry into the tissues is critical to understand the energy load of detoxification or confrontation of $O_3$ for the plant and that load effect on the plant's productivity.

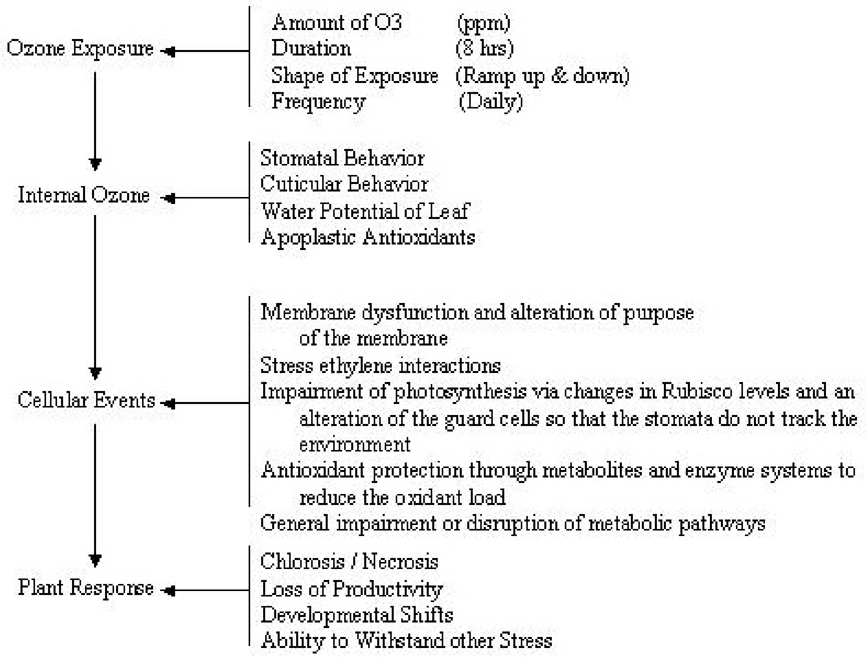

**FIGURE 1.** Sequence of ozone interaction with plant tissues. A shortened version of the important events with ozone interacting with leaf tissue. Some of the events shown here are just touched on, while others are discussed in the text at length. This is a short roadmap of important measures.

## TABLE 1
## Some of the Up-Regulated Genes from *Arapidopsis thaliana* due to Ozone Exposure

| Annotation | AGI Code | Col-0 | rcd1 |
|---|---|---|---|
| Pathogenesis-related protein-1 (PR-1) | At2g14610 | 65 | 69.1 |
| Plastocyanin-like domain-containing protein | At5g20230 | 63.1 | 47.2 |
| Glutathione S-transferase, putative | At4g02520 | 26.8 | 63.9 |
| Glutathione S-transferase, putative | At1g02930 | 18.1 | 24.8 |
| Hevein-like protein | At3g04720 | 10.3 | 30.8 |
| Legume lectin family protein | At3g15350 | 10.2 | 15.3 |
| Plant defensin (PDF1.2a) | At5g44420 | 7.8 | 3.1 |
| Basic chitinase | At3g12500 | 7.8 | 20.3 |
| Peroxidase, putative | At3g49120 | 7.2 | 5.6 |
| Peroxidase | At5g39580 | 5.1 | 4.4 |
| Defender against death1 (dnd1) | At1g32210 | 4.3 | 3.4 |
| Trp synthase -subunit | At5g54810 | 3.7 | 5.1 |
| Wall-associated kinase1 (WAK1) | At1g21250 | 2.9 | 7.7 |
| ACC oxidase 2 | At1g62380 | 2.2 | 5.4 |
| Glutathione S-transferase 6 (GST6) | At2g47730 | 2 | 3.9 |

The samples were taken 8 h after the beginning of a 6-h $O_3$ exposure of 250 nl/l. The values depict the average ratios of m-RNA abundance between $O_3$-treated and clean-air-grown material from two biological repeats. The data from the macroarray were first normalized to the mean of m-RNA abundance of actin genes *ACT2* (At3g18780) and *ACT8* (At1g49240), which were shown to be expressed constitutively by RNA gel blots. Many other signals are called "putative". A complete list of the genes used can be seen at www.helsinki.fi/biosci/plantstress/contents/publications/macroarray.html.

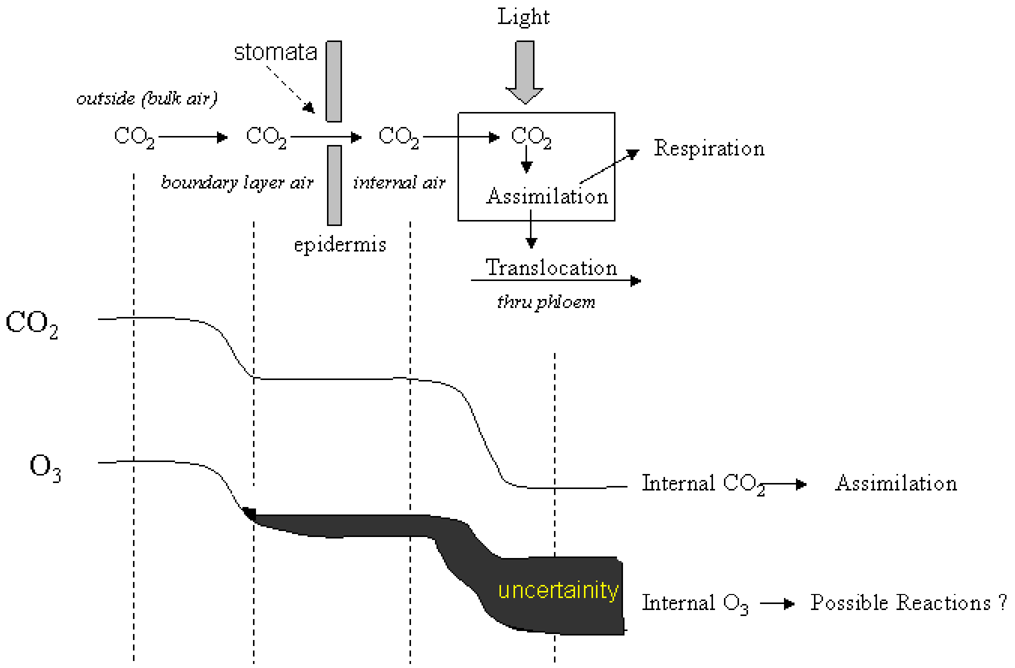

**FIGURE 2**. Pathway and relative concentration of ozone and $CO_2$ movement into leaf tissue. The pathways of both gases are shown left to right following the structures presented above. The concentrations of the gases are shown in relative terms up and down, with the higher level representing the higher concentration. It should be noted that there is an uncertainty of the level of ozone within the leaf due to the types of reactions that ozone may undertake. No measurement has been made of ozone within the leaf, rather either breakdown of ozone when exposed to leaf tissue[7] or the loss of ozone that pass through the leaf[8] are measured.

There are five general concepts that have been developed since the 1960s regarding the plant's response to ozone at a cellular level (given in Fig. 1, middle). Of these, the concept of a general impairment is the most general, but has the potential to yield great insight into protection from ozone, if we can better define this somewhat vague term.

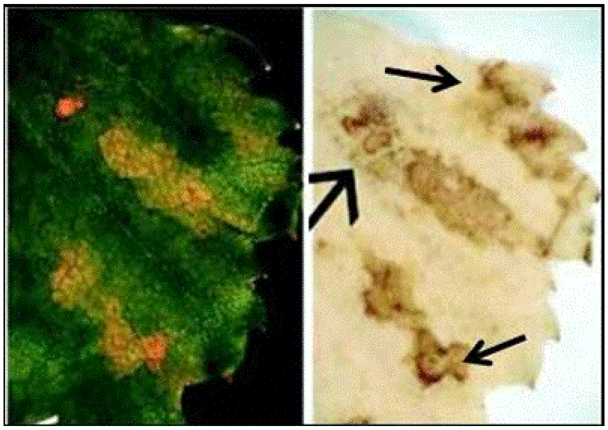

**FIGURE 3.** Accumulation of $H_2O_2$ within ozone-exposed leaf tissue. Lesion development and $H_2O_2$ accumulation in birch leaves. Taken and modified from Fig. 1[9]. Birch leaf with lesions 10 h after the beginning of an 8-h ozone (150 nl/l) exposure. Left, normal photograph; right, DAB staining reveals $H_2O_2$ accumulation at and around lesion sites (arrows) in a cleared leaf 2 h later.

During earlier times of investigation, studies examined individual proteins whose activity could be measured. Furthermore, for long periods of time, very high levels of ozone were typically used to induce measurable changes. This meant that many of the studies were on proteins that may not have been important or on nonimportant proteins that were increased or decreased only by excessively high concentrations of ozone. Thus, many of these early studies do not really reflect what happens during general impairment under realistic exposures of ozone. A few enzymes were shown to change and still seem to be important today. Those are what can be deemed to be "real changes" of the impairment concept induced by ozone (those underlined below and emphasized later). A partial listing of the studied enzymes are phosphoglucomutase[13], peroxidase and cellulose[14], nitrite reductase[15], glyceraldehyde-3P dehydrogenase, glucose-6P dehydrogenase, phenylalanine ammonia lyase (PAL), polyphenol oxidase and peroxidase[16], glucose-6P dehydrogenase, isocitrate dehydrogenase, alanine aminotransferase and glutamate dehydrogenase[17], and superoxide dismutase, β-1,3-glucanase, chitinase, pathogenesis-related proteins (PR), lipoxygenase, glutathione reductase and ascorbate peroxidase (summary by Harris and Bailey-Serres[18]).

The most carefully studied enzyme that is influenced by ozone is Rubisco[19,20,21] (Fig. 4). These studies found that ozone lowered assimilation under conditions with minimum change in stomatal conductance due to a loss of Rubisco activity, which ultimately was found to be equivalent to early senescence. That loss of Rubisco was due to a decline in the m-RNA of the enzyme, in particular due to decline of *rbsS* (RNA for the small subunit). The lower production of m-RNA from one or more isoforms of DNA was due to a change of transcriptional factor (not yet identified) induced by a change in signal transduction. This was the first and best case of the tracing of the loss of a function (assimilation) to the loss of an enzyme (Rubisco) to the decline of the message for it (*rbsS*) by some signal transduction. Some of the signals[21] were shown to be equivalent to those due to senescence (e.g., SAGs)

One of the more recent discoveries of an enzyme change induced by $O_3$ is for glutathione-sulfhydryl-transferase (GST)[22], which can reduce a wide array of compounds and is linked to the regeneration of reducing power within the cell such as ascorbate. The important point of these studies is that the GST message — there may be more than one isoform of GST, each of which may be induced by a different event — is stimulated within hours of exposure, but then its level drops over the course of a day taking about 10 h to decline to a 50% level. This rapid rise and subsequent fall is not completely repeated on repeated exposures. For example, the amount after 6 h of exposure falls the second day and then stabilizes after about 3–4 days at a 50% level. If other messages are examined, they may have quite different time sequences (for example, see an ethylene enzyme such as ACC[23]).

There are three principle methods of determining what sort of metabolic events are occurring: (1) measure the activity of a particular enzyme after opening the cell (velocity), (2) measure the amount of each protein that is expressed within the tissue (mass), and (3) measure the messages for the construction of each protein (m-RNA level). The early days of investigation used either method 1 or 2. The activity 1 is problematic since the enzyme itself can be heavily regulated and so one may measure a false activity due to a change in regulation when the enzyme is released from the cell. Measurement 2 suffers from a similar, but inverse, problem; the amount may not be proportional to the speed that the enzyme may convert substrate to product.

Many have turned to measurement 3, the expressed m-RNA present in the cell during a certain developmental time period of the tissue. This certainly is a measure of how much protein may be expressed, providing the protein is not turned over or degraded rapidly. Yet, it is still only an idea of how much enzyme is expressed within the cell. Comparison between ozone-exposed and control tissue may be useful as to the expression of a particular message from the DNA informational base. The measurement of m-RNA can take on several forms, but the use of probes of a particular opposite-sense m-RNA or a fragment of the DNA that coded for the RNA locked onto a membrane has been very useful — the microarray. The expressed m-RNA binds to the probe and, in doing so, changes some sort of indicator, often a chemical fluorescence signal due to the covering up of the probe by the expressed message.

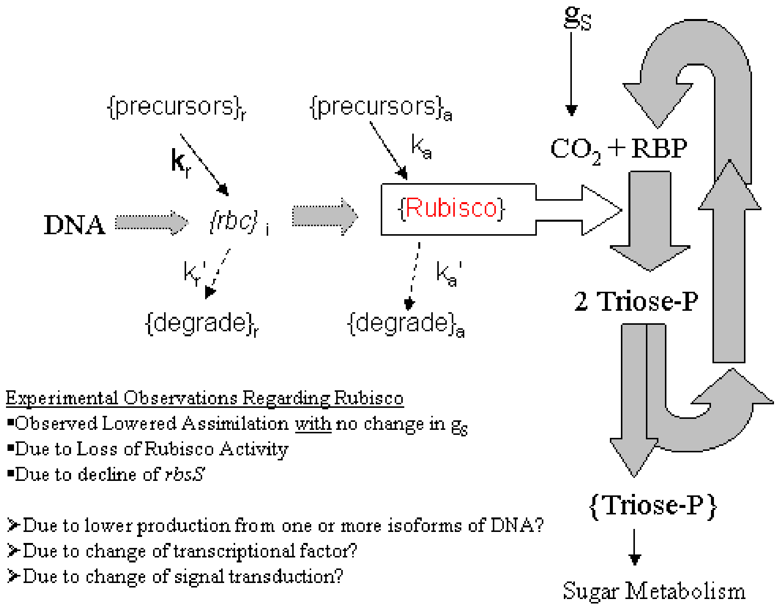

**FIGURE 4.** The production of Rubisco and its Calvin Cycle pathway reactions. Schematic of the movement of information from DNA (starting at the left) to the production of the protein, Rubisco (ribulose 1,5-bisphosphate carboxylase/oxygenase), and its integration into the Calvin Cycle (cyclic arrows on right). $CO_2$ levels are controlled by the stomatal conductance ($g_S$). The sequence of changes is from Pell's group[20,21].

The probes on the membrane may be also c-DNA, which are fragments generated from known DNA sequences of genes. Such a c-DNA chip for ozone-exposed *Arabidopsis* leaves[24] demonstrates such expression due to ozone. In this case, green spots represent genes up-regulated (more m-RNA than the control), yellow spots represent genes of equal or similar expression, and red spots represent down-regulation of genes (less m-RNA than in the control). While the technology is currently quite good, a probe of the actual m-RNA expressed is required and a limit of change must be determined for which a real effect is alleged (generally changes in concentration of two to three times). Fig. 5 shows the plot of expression for a large number of EST on a microarray (expressed sequence tags, c-DNA from expressed genes via RNA messages[25]) for normal leaves and leaves infiltrated with $H_2O_2$. Most of the EST are not changes, but depending on the deviation from linearity that is taken, we can obtain a large number. More importantly, there are some differences that are clear, as shown by the circled regions.

In the data from Matsuyama et al.[24], a strong green signal was observed for c-DNA sequences of PR-1, PR-2a, and PR-5; these are the pathogen-response genes induced by a pathogen attack on the leaf. Also noted were changes for the GST as before, but only for two isoforms, GST1 and GST2. A strong response was noted for EDS1 (representing disease resistance protein, gene name T17F15.40). A weak green repression was detected for PR-3b and PR-4. These responses follow some of the responses noted before. Not all possible genes are represented on most plates; in this case, there were only 196 genes on the plates out of some 20,000 genes from *Arabidopsis*. As the authors stated, "Expression profiles revealed that we could discern $O_3$-stress from the responses induced by drought- or wounding-stress while we could not discriminate between $O_3$-stress and UV-B-stress mRNA." This result can be obtained due to overlapping pathway responses or not enough probes to clearly discern differences.

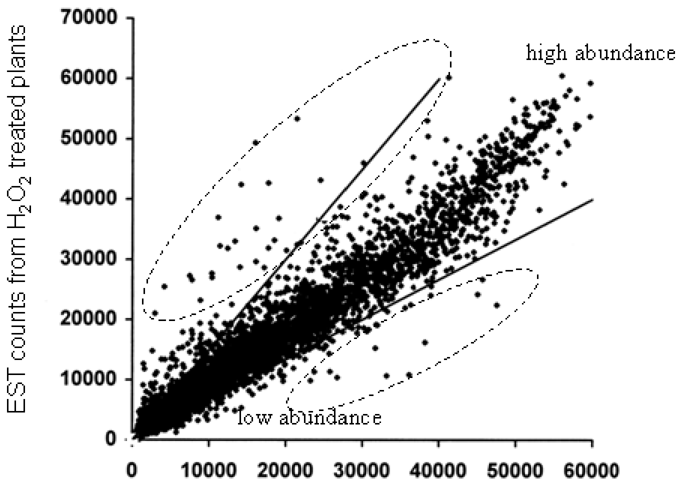

**FIGURE 5.** Scatter plot of signal intensities for all EST (expressed sequence tags) on the microarray. Normalized channel intensities for each clone on the microarray are plotted with signals from control and $H_2O_2$-treated cells. The diagonal lines represent 1.5-fold induction/repression ratio cutoffs. The ovals represent gene expression that is clearly up-regulated (top oval) or down-regulated (bottom oval). Modified from Desikan et al.[25].

There are other current problems with this approach. We simply do not know enough yet about each gene and its control elements' up-translation direction from the promoter region. Table 1 demonstrates some of the uncertainty as well as other changes observed in m-RNA probes. The samples were harvested 8 h after the beginning of a 6-h $O_3$ exposure of 250 nl/l. The values represent the average ratios of m-RNA abundance between $O_3$-treated and clean-air-grown material. Several messages of the classical proteins are observed, such as some isoforms of GST, ACC oxidase, a number of peroxidases, PR protein, chitinase, and a few new proteins (DND, L-idonate 5-dehydrogenase, and WAK, wall-associated kinase). Here is another good example of how not all isoforms (different regions of the DNA expressing the same gene) are stimulated. More interestingly, there are some activated genes of enzymes required in different pathways that have previously been observed, such as tryptophan synthase and plastocyanin-domain proteins. These results may lead us into a different set of experiments regarding why they should be expressed and how these pathways may have been previously implicated.

Returning to the experiments of Desikan et al.[25], which examined the expression of genes generated by infiltrating $H_2O_2$ into leaves, we find some similar results, but with important differences. The data from this chip were normalized to the mean of m-RNA abundance of two actin genes (*ACT2* and *ACT8*). Aside from the two previously found changes due to ozone exposure (GST and nitrite reductase), only a few known m-RNA are up-regulated (cytochrome P450, a cytochrome used for foreign molecule redox reactions), several heat-shock proteins (hsp), glutamyl transferase, blue Cu-binding protein, and a Ca-binding protein. The majority of the expressed genes are for unknown proteins or proteins of only putative activities out of the 176 total up-regulated proteins.

Similar experiments of Cheong et al.[26] generated changes in messages induced by several forms of stress. The number of m-RNA forms that were up-regulated was large, with 129 expressed genes from wounding, 94 from pathogen, 44 from abiotic stress, and 28 from hormonal response. Which messages overlap those observed for ozone? Interestingly, those involved with ethylene (ACC synthase) and peroxidase were the most similar to ozone-induced stress.

In the real world, ozone is not the only air pollutant present and many past experiments have attempted to show effects with several stressors given simultaneously. One experiment, which proved to be interesting, involved the application of two stresses at the same time compared with the application of only one at a time[27]. There was an enhanced expression of some 1,075 genes induced by drought and of some 262 from heat alone. However, when both heat and drought were combined, there were 1,057 genes with enhanced expression. Of them, 450 overlapped those from only drought and 124 overlapped those from only heat. Only 29 of those from the combined stress were expressed in both drought and heat alone. Thus, only 60% of those expressed with the combined stress were the same as either stress alone. More importantly, finding expressed genes in both heat and drought stress demonstrates only a few of the genes (29) that are expressed with combined stresses. That means that simple experiments with only one exposed stress at a time cannot be used to give a true picture of what is occurring under multiple stresses.

So how should we begin to think about cellular events on ozone exposure? After ozone enters the leaf tissue, it first reacts with extracelluar antioxidants and that interaction seems to stimulate ascorbate production and/or its movement from one compartment to another. The production of $H_2O_2$ next to the membrane seems to both trigger a pathogen-like response and generate more $H_2O_2$, which may lead to a "runaway" feedback situation. That response gives rise to PR proteins linked to pathogen attack and leads into a wound-like signal, involving ethylene, peroxidases, and inducing changes in cell wall formation. Physical wounding involves some of these responses, notably ethylene release, but also involves both Ca changes within the cytoplasm and other membrane transport alterations, leading to a localized spread of any injury and the formation of systemic signals, such as salicylic acid[28].

This concept is an interesting beginning, but interactions of varied signal transduction pathways have been strongly suggested by current experiments, and those linkages need to be understood and well explored. However, it must be remembered that ozone attack may not signal an ozone-specific metabolic change. There are many pathways to the final visible injury or lowering of productivity that we observe.
In particular, ozone has been shown to partially close stomata and a lowered conductance means less assimilation, which means less sugar within the tissue. Less sugar means a change in sugar regulation and that would be expected to change the sugar transduction signals such as SGU. Furthermore, less sugar means accelerated senescence signals, such as *AP2* and *EREBPs*. Also lowered conductance means less water loss from the leaves and that would mean higher leaf water potential, leading to less drought signaling. While ozone has been shown to generate local regions of $H_2O_2$, a higher localized level of $H_2O_2$ has been shown to alter stomatal conductance directly, such as through the signal ABI[29].

So which alterations of biochemical pathways of plants are the true gauges of injury? Currently, the list is short with extracellular pools and flows of ascorbate, and the use of ethylene as markers of "some kind of injury" along with the classical enzyme systems, such as Rubisco, ACS, peroxidases, and PAL. But all must be used with caution, using a good definition of the actual exposure used, and species and developmental age of the plant. Do not expect a "magic microarray" quite yet; however, those chips with wounding injury, oxidative exposure, and pathogen-response c-DNA or m-RNA elements may be quite useful. We have come a long way since the mid-20$^{th}$ century, but still have a long way to go. Luckily, research into general stress responses that we can piggy-back onto is making great progress.
